# A naturalistic neuroimaging database for understanding the brain using ecological stimuli

**DOI:** 10.1038/s41597-020-00680-2

**Published:** 2020-10-13

**Authors:** Sarah Aliko, Jiawen Huang, Florin Gheorghiu, Stefanie Meliss, Jeremy I. Skipper

**Affiliations:** 1 London Interdisciplinary Biosciences Consortium, University College London, London, UK; 2 Experimental Psychology, University College London, London, UK; 3 School of Psychology and Clinical Language Sciences, University of Reading, Reading, UK

**Keywords:** Cognitive neuroscience, Databases

## Abstract

Neuroimaging has advanced our understanding of human psychology using reductionist stimuli that often do not resemble information the brain naturally encounters. It has improved our understanding of the network organization of the brain mostly through analyses of ‘resting-state’ data for which the functions of networks cannot be verifiably labelled. We make a ‘*Naturalistic Neuroimaging Database*’ (NNDb v1.0) publically available to allow for a more complete understanding of the brain under more ecological conditions during which networks can be labelled. Eighty-six participants underwent behavioural testing and watched one of 10 full-length movies while functional magnetic resonance imaging was acquired. Resulting timeseries data are shown to be of high quality, with good signal-to-noise ratio, few outliers and low movement. Data-driven functional analyses provide further evidence of data quality. They also demonstrate accurate timeseries/movie alignment and how movie annotations might be used to label networks. The NNDb can be used to answer questions previously unaddressed with standard neuroimaging approaches, progressing our knowledge of how the brain works in the real world.

## Abstract (summary)

**Table:** 

Measurement(s)	functional brain measurement • Behavioral Assessment
Technology Type(s)	functional magnetic resonance imaging • NIH Toolbox
Factor Type(s)	movie
Sample Characteristic - Organism	Homo sapiens
Sample Characteristic - Environment	laboratory facility

Machine-accessible metadata file describing the reported data: 10.6084/m9.figshare.12869855

## Background & Summary

A primary goal of human neuroscience is to understand how the brain supports broad psychological and cognitive functions that are engaged during everyday life. Progress towards achieving this goal over the last two decades has been made with tens of thousands of task- and resting-state based functional magnetic resonance imaging studies (henceforth, task-fMRI and resting-fMRI). While these studies have led to a number of important discoveries, we review evidence suggesting that a better understanding of brain and behaviour might be achieved by also conducting studies with more ecologically valid stimuli and tasks (naturalistic-fMRI).

### Task-fMRI

For task-fMRI, general psychological processes are decomposed into discrete (though hypothetical) component processes that can theoretically be associated with specific activity patterns. To ensure experimental control and because of reliance on the subtractive method^1^, these components are studied with stimuli that often do not resemble things participants might naturally encounter and tasks they might actually perform in the real-world (a topic long debated)^2–4^. For example, language comprehension has been broken down into component processes like phonology and semantics. These individual subprocesses are largely localised in the brain using isolated auditory-only ‘speech’ sounds (like ‘ba’) in the case of phonology and single written words in the case of semantics^5^. Participants usually make a meta-linguistic judgement about these stimuli, with a corresponding button response (e.g., a two-alternative forced choice indicating whether a sound is ‘ba’ or ‘pa’). This is not peculiar to the study of language comprehension. For example, neuroimaging studies of emotional processing usually use static pictures of faces making exaggerated emotional displays and require, e.g., a button press if the face is female^6^.

The result of relying on these ‘laboratory style’ stimuli and tasks is that our neurobiological understanding derived from task-fMRI may not be representative of how the brain processes information. This is perhaps one reason why fMRI test-retest reliability is so low^7,8^. Indeed, more ecologically valid stimuli like movies have higher reliability than resting- or task-fMRI. This is not only because they decrease head movement and improve participant compliance^9–11^. Rather, naturalistic stimuli have higher test-retest reliability mostly because they are more representative of operations the brain normally performs and provide more constraints on processing^12–17^.

### Resting-fMRI

There has arguably been a significant increase in our understanding of the network organization of the human brain because of the public availability of large resting-fMRI datasets, analysed with dynamic and other functional connectivity methods^18,19^. These include the INDI ‘1000 Functional Connectomes Project’^20^, ‘Human Connectome Project’ (HCP)^21^ and UK Biobank^22^. Collectively, these datasets have more than 6,500 participants sitting in a scanner ‘resting’. Resulting resting-state networks are said to represent the ‘intrinsic’ network architecture of the brain, i.e., networks that are present even in the absence of exogenous tasks. These networks are often claimed to be modular and to constrain the task-based architecture of the brain^23^.

As with task-fMRI, one might ask how representative resting-state networks are given that participants are anything but at rest. They are switching between trying to fixate a cross-hair, staying awake, visualising, trying not to think and thinking through inner speech^23,24^. Though some of these behaviors are ‘natural’, unlike task-fMRI, there is no verifiable way to label resulting regional or network activity patterns^25,26^. At best, reverse inference is used to give 5–10 gross labels, like the ‘auditory’ and ‘executive control’ networks^27–29^. Despite claims that these ‘intrinsic’ networks constrain task-fMRI networks, it is increasingly suggested that this is not necessarily so^23^. The brain is less modular during task- compared to resting-fMRI^30^ and modularity decreases as tasks get more difficult^31–33^. Indeed, up to 76% of the connections between task- and resting-fMRI differ^34^. Furthermore, more ecological stimuli result in *new* sets of networks that are less modular and only partly constrained by resting networks^35,36^.

### Naturalistic-fMRI

Based on considerations like these, there is a growing consensus that taking a more ecological approach to neuroscience might increase our understanding of the relationship between the brain and behaviour^5,37–46^. This includes conducting more neuroimaging studies with ‘naturalistic’ stimuli. Similar to prior definitions^25,47^, ‘naturalistic’ might be defined on a continuum from short, static, simple, decontextualised, repeating, unisensory stimuli with low ecological validity (as described above) to long, dynamically changing, complex, contextualised, continuous, typically multisensory stimuli with high ecological validity. At the time of writing, there were at least 16^48^ (and growing^49^) publicly available fMRI datasets using ‘naturalistic’ stimuli more on the latter end of this continuum. However, there are no datasets with a large number of participants, long naturalistic stimuli and stimulus variability. Specifically, most datasets have a small number of participants (median = 23). However, 80 or more participants are preferred for detecting medium effect sizes and producing replicable task-fMRI results^50–52^. The naturalistic-fMRI datasets with larger numbers tend to use short (~10 minute) audio or audiovisual clips. However, stimulation times of 90 minutes or more are preferred for reliability and individual analyses^53–56^.

Longer duration fMRI datasets using more naturalistic stimuli have a small number of participants and one stimulus (though see^57^). These include 11 people watching ‘Raiders of the Lost Ark’^58^ and 20 listening to an audio description of ‘Forrest Gump’ during fMRI. A subset of the latter returned to be scanned watching the movie dubbed in German (http://studyforrest.org)^59,60^. However, with only one movie, generalisability is limited. More movies would not only increase generalisability, they would increase the number of stimulus features and events in a variety of (jittered) contexts that might be annotated. These could then be used to label finer grained patterns of activity, e.g., making machine learning/decoding approaches more feasible^61–63^.

Indeed, there is no a priori reason participants need to watch the same movie (or listen to the same audio). Existing long datasets might use one stimulus because intersubject correlation is a commonly used method for analysing fMRI data from more naturalistic stimuli that are difficult to model^64^. Though this is a powerful ‘model-free’ approach (for an overview, see^65^), it requires participants to watch the same movie. However, many, if not most, questions are stimulus-feature or event specific and independent of the movie being watched. Thus, ‘model-free’ (more data-driven) methods like independent component analysis^66^, regional homogeneity^67^, hidden markov model^68^ and dynamic mode decomposition^69^ and more model-based analysis involving convolution/deconvolution, can be done at the individual participant level with *different* movies. This would increase generalisability and the possibility of more detailed analyses through more varied stimulus annotations.

### NNDb

**Fig. 1:**
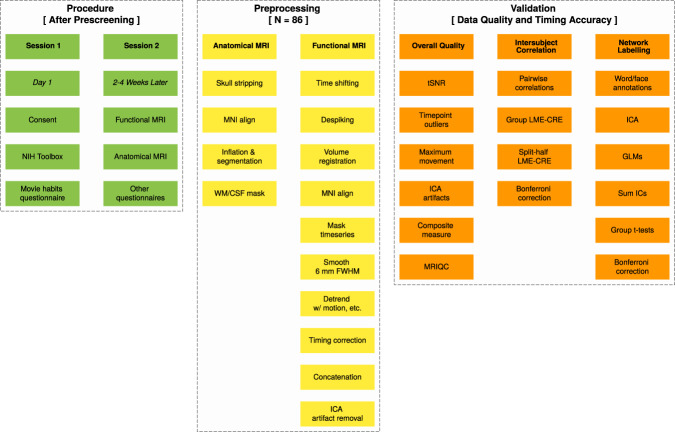
Schematic overview of the *naturalistic neuroimaging database* procedures, preprocessing and data validation. Procedures (green) occurred over two sessions separated by about three weeks on average. Session one consisted primarily of a battery of behavioural tests to quantify individual differences. In session two, functional magnetic resonance imaging (MRI) was acquired while participants watched one of 10 full length movies followed by anatomical MRI (see Tables 1 and 2). After preprocessing the data (yellow), three primary data validation approaches were undertaken (orange). The fMRI data is shown to be relatively free of outliers, with good temporal signal-to-noise ratio (tSNR) and low numbers of outlier timepoints, head movement and independent component analysis (ICA) artifacts. Data quality was also verified using MRIQC software for extracting image quality metrics (orange, column 1; see Tables 4–6 and Fig. 2). Intersubject Correlation analyses provide evidence for functional data quality and the temporal synchronization between participants and movies using linear-mixed effects models with crossed random effects (MNE-CRE; orange, column 2; see Fig. 3). Automated word and face annotations were used to find associated independent component (IC) timecourses from ICA using general linear models (GLMs; orange, column 3; see Tables 3 and Fig. 4). In addition to further illustrating data quality and timing accuracy, this analysis shows how annotations might be used to label brain networks. See Online-only Table 1 for the location of all data and scripts/code associated with this manuscript.

To fill these gaps in publicly available data, we created a ‘*Naturalistic Neuroimaging Database*’ (NNDb) from 86 people who each did a battery of behavioral tests and watched a full-length movie during movie naturalistic-fMRI. We sought to reach a balance that promotes generalizability, allows a large variety of stimulus features and events to be annotated and permits the use of intersubject correlation and the other analyses described above. To achieve this, our participants watched 10 different movies from 10 different genres. They had not previously seen the movies they watched because multiple viewings might change the functional network architecture of the brain (though activity patterns may appear similar)^70^. We validate that the data is of a high quality and good temporal alignment, whilst providing an example of using annotations to label networks. Figure 1 and Online-only Table 1 provides an overview of the study and analyses used to make this assessment.

Data discovery is nearly unlimited with the NNDb as there are a vast number of annotations that can be made from the movies and approaches to analysis. This flexibility makes it usable across disciplines to address questions pertaining to how the brain processes information associated with more naturalistic stimuli. This includes more than replicating prior findings with more ecologically valid stimuli. That is, there are a number of broad open questions that the NNDb can be used to address for the first time, like the systematic study of how context is used by the brain^5^. Given the lack of robust neuroimaging biomarkers for mental illness^71,72^, the NNDb might also help increase the pace of clinically relevant discovery, e.g., by uncovering labelled network patterns that predict individual differences^46^.

## Methods

### Participants

Our goal for the NNDb v1.0 was to create an initial dataset with 84 participants watching 10 full-length movies from 10 genres. Specifically, we set out to collect fMRI data from 18 people watching two movies and six people each watching eight additional movies. This was roughly based on sample size considerations reviewed above^50–56^, the desire for stimulus variability and methodological considerations. That is, we reasoned that 84 participants should be sufficient to power most analysis with a set of features across all movies. Two larger datasets would allow those who do not want to work across movies to do hypothesis testing and generalisation with a typical number of participants. Eight additional datasets would allow for analyses with greater stimulus variability and generalisability with what we considered a minimum number of participants per movie (e.g., to do intersubject type analyses). Nonetheless, this sample size is somewhat arbitrary as we cannot predict what analysis different groups might do. Furthermore, we plan to continue data collection, having more participants watch more movies.

To reach 84 individuals, we identified 120 possible recruits using participant pool management software (http://www.sona-systems.com/). These recruits were screened for MRI safety (e.g., no metal implants) and inclusion criteria. The latter required that participants be right-handed, native English speakers, with no history of claustrophobia, psychiatric or neurological illness, not taking medication, without hearing impairment and with unimpaired or corrected vision. We also pseudo-randomly selected participants meeting these criteria to assure that they had not seen one of the 10 movies and so that the final sample was relatively gender balanced. Thus, of the 120 recruits, 91 met these contingencies, were enrolled and completed the study. We collected more than 84 participants under the assumptions that some number would need to be excluded as data quality outliers. Indeed, two were excluded as they were determined to be left handed afterall, two because they asked to get out of the scanner multiple times and one who had low data quality. This left us with two additional participants than we had set out to collect.

The final sample consisted of 86 participants (42 females, range of age 18–58 years, M = 26.81, SD = 10.09 years). These were pseudo-randomly assigned to a movie they had not previously seen, (usually) from a genre they reported to be less familiar with. Table 1 provides a summary of participant demographics by movie. At the conclusion of the study, participants were given £7.5 per hour for behavioural testing and £10 per hour for scanning to compensate for their time (receiving ~£40 in total). The study was approved by the ethics committee of University College London and participants provided written informed consent to take part in the study and share their anonymised data.

**Table 1:** Description of participants in the *naturalistic neuroimaging database*.

N	Movie	Age	%			≥Bachelor’s (%)		NIH Toolbox Examples	
			Female	BAME	Monolingual	Participant	Mother	Fluid Cog	Neg Affect
20	500 Days of Summer	27.70	50.00	30.00	85.00	75.00	60.00	58.11	51.25
18	Citizenfour	27.00	50.00	41.18	77.78	61.11	66.67	53.71	51.22
6	12 Years a Slave	27.17	50.00	66.67	50.00	50.00	50.00	31.00	50.83
6	Back to the Fuure	22.17	50.00	40.00	66.67	66.67	83.33	39.67	57.67
6	Little Miss Sunshine	35.67	33.33	66.67	66.67	50.00	16.67	45.67	52.33
6	The Prestige	34.17	50.00	0.00	100.00	83.33	33.33	76.00	51.67
6	Pulp Fiction	22.67	50.00	83.33	66.67	33.33	0.00	51.00	57.67
6	The Shawshank Redemption	22.17	50.00	100.00	50.00	50.00	83.33	68.00	50.00
6	Split	22.67	50.00	50.00	83.33	66.67	33.33	52.67	57.50
6	The Usual Suspects	23.17	50.00	66.67	83.33	100.00	33.33	55.33	54.50
86	wMean	26.73	48.84	48.62	75.58	65.12	51.16	54.01	52.79
	wSD	3.99	4.27	24.62	13.48	15.99	23.49	10.47	2.67

All participants (N) were right-handed and native English speakers. Gender is expressed as percent female. Ethnic diversity is expressed as percent Black, Asian and Minority Ethnic (BAME). Educational attainment of both the participant and the participant’s mother is expressed as percent with a Bachelor’s degree or higher. Data roughly match 2011 London, UK consensus data (https://data.london.gov.uk/census/). We include the Cognition Fluid Composite v1.1 (Fluid Cog) and Negative Affect Summary (18+)(Neg Affect) T-scores as example tests from the National Institute of Health (NIH) Toolbox battery. The bottom two rows are the means and standard deviations of row means weighted by number of participants (wMeans/wSD).

### Procedures

Participants meeting inclusion criteria were scheduled for two sessions on seperate days. During session one, participants gave informed consent and then completed the majority of the National Institute of Health (NIH) Toolbox. This provides demographic data and validated measures of sensory, motor, cognitive and emotional processing that might be used as individual difference measures^73^. We only excluded tests in the ‘Sensation’ and ‘Motor’ domains that required physical implementation (e.g., scratch and sniff cards, a pegboard, endurance walking, etc.). Participants were provided with headphones and tests were administered in a sound shielded testing room on an iPad. At the end of session one, participants filled out a questionnaire on movie habits, including information on preferred movie genres. The entire session typically took about one hour.

Functional and anatomical MRI and a final questionnaire were completed during a second session that was separated from the first by about 2–4 weeks (M = 20.36 days; SD = 23.20). Once in the scanning suite, participants reporting corrected vision were fitted with MRI-safe glasses. They chose earbud sizes for the noise-attenuating headphones that were comfortable. Next, participants were put in the head-coil with pillows under and on the sides of their head and under the knees for comfort and to reduce movement over the scanning session. Once in place, participants chose an optimal stimulus volume by determining a level that was loud but comfortable. Video presentation was adjusted for optimal viewing quality. Participants were given a bulb in their right hand and told to squeeze if something was wrong or they needed a break during the movie. They were instructed to not move as best as they could throughout scanning as movement would make the scans unusable.

Except in one case, fMRI movie scans were acquired first and with as few breaks as possible. During breaks, participants were told that they could relax but not move. During scanning, participants were monitored by a camera over their left eye. If they appeared drowsy or seemed to move too much during the movie, the operator of the scanner gave them a warning over the intercom by producing a beep or speaking to them. In some cases we stopped the scan to discuss with the participant. After the movie, participants had an anatomical scan and were told they could close their eyes if they wished. Following scanning, participants filled out other questionnaires, e.g., about their specific experience with content in the movie they watched. Finally, participants were paid and sent home.

### Movie stimuli

Table 2 provides an overview of the 10 movies participants watched during fMRI and information on how to purchase these (so that they can be used to create new annotations). The movies were chosen to be from 10 different cinematic genres and to have an average score of >70% on publicly available metrics of success. These were the *Internet Movie Database* (IMDb; https://www.imdb.com/), *Rotten Tomatoes* (RT; https://www.rottentomatoes.com/) and *Metacritic* (https://www.metacritic.com/).

**Table 2:** Description of the movies used in the *naturalistic neuroimaging database*.

Movie	Genre	Year	Length (s)	Scores (%)			DVD Version	
				IMDB	Meta	RT	ASIN	EAN
500 Days of Summer	Romance	2009	5470	77	76	85	B002KKBMSW	5039036043359
Citizenfour	Documentary	2014	6804	81	88	96	B00YP65NEI	5050968002313
12 Years a Slave	Historical	2013	7715	81	96	96	B00HR23CCM	5030305517229
Back to the Future	Sci-fi	1985	6674	85	86	96	B000BVK82I	5050582401288
Little Miss Sunshine	Comedy	2006	5900	78	80	91	B000JU9OJ4	5039036029667
The Prestige	Thriller	2006	7515	85	66	76	B000K7LQS8	7321902106472
Pulp Fiction	Action	1994	8882	89	94	94	B004UGAMY4	5060223762043
The Shawshank Redemption	Drama	1994	8181	93	80	91	B001CWLFKE	5037115299635
Split	Horror	2016	6739	73	62	76	B071J24232	5902115603099
The Usual Suspects	Crime	1995	6102	86	84	95	B0010YXNGI	5039036033497

Ten full length movies were chosen from 10 genres. These were required to have been successful, defined as an average *Internet Movie Database* (IMDb), *Metacritic* (Meta) and *Rotten Tomatoes* (RT) score greater than 70%. IMDb scores were converted to percentages for this calculation. Movie lengths are given in seconds (s), also equivalent to the number of whole brain volumes collected when participants watched these movies during functional magnetic resonance imaging. The DVD version of the movies used in the database can be purchased with their unique Amazon Standard Identification Number (ASIN) or International/European Article Number (EAN).

All movies were purchased and stored as ‘.iso’ files. Relevant sections of the DVD (i.e., excluding menus and extra features) were directly concatenated to a mpg container using:

*ffmpeg -i concat:VTS_01_1.VOB\|... VTS_01_8.VOB -c copy -f dvd movie_name.mpg*

where ‘-c’ copies the codec and ‘-f’ specifies the DVD format. This generally maintains the original DVD video size and quality, using all frames with no cropping or other transformations:

- Video (codec): MPEG-PS
- Audio (codec, sampling rate, bits per sample, channels): AC-3, 48.0 kHz, 16, 6
- Resolution (pixels): 720 × 576 (except Citizenfour which was 720 × 480)
- Aspect Ratio: 16:09 (except The Usual Suspects and Pulp Fiction which were 2.40:1 4:3, respectively)
- Frame rate (fps): 25 (except Citizenfour which was 23.976)

The resulting files were presented to participants in full-screen mode through a mirror reversing LCD projector to a rear-projection screen measuring 22.5 cm × 42 cm with a field of view angle of 19.0°. This screen was positioned behind the head coil within the MRI bore and was viewed through a mirror above participants’ eyes. High quality audio was presented in stereo via a Yamaha amplifier through Sensimetrics S14 scanner noise-attenuating insert earphones (https://www.sens.com/products/model-s14/).

#### Movie pausing

Movies were played with as few breaks as possible. This allows for the most natural, uninterrupted viewing experience and minimises the number of discontinuities in the hemodynamic response. It also results in good timing accuracy, needed for relating movie features and events to brain responses. It maintains timing by avoiding unknown and accumulated human and hardware processing delays associated with starting and stopping. To accomplish continuous play with the possibility of arbitrary stopping points, we created a script and hardware device to allow the operator to stop the scanner and pause the movie at any time and resume where the movie left off when the scanner was restarted. Unless participants signalled that they wanted a break, the movies were played in about 40–50 minute segments (because of a software limitation on the EPI sequence we used). These breaks were timed to occur during scenes without dialogue or relevant plot action.

Specifically, a Linux BASH script opened and paused movies using ‘*MPlayer*’ (http://www.mplayerhq.hu/). The script then went into a state of waiting for a TTL (transistor-transistor logic) pulse from the scanner, indicating that scanning had begun. Pulses were received through a USB port connected to an Arduino Nano built to read and pass TTL pulses from the scanner to the script. When the scan was started and the first TTL pulse was received, eight seconds were allowed to elapse before the movie began to play. These timepoints allowed for the scanner to reach a state of equilibrium and were later discarded. If the scanner was subsequently paused, e.g., because the participant requested a break, the movie pausing BASH script stopped the movie within 100 ms. This known delay occurred because the script monitors for TTL pulses every 50 ms. If a pulse was not registered, the script required that the next pulse also did not arrive before pausing to assure pulses had stopped. When the scan was restarted, eight seconds were again allowed to pass before the movie was unpaused.

Whenever a movie was paused after it had been playing, the whole brain volume being collected was dropped, causing up to one second of the movie to be lost from the fMRI timeseries. There were two versions of the script. In the first, the movie picked up where it left off when it had been paused (v1; N = 29 or 33.72% of participants). The second version rewound the movie to account for the time lost from the dropped volume. To calculate this, the script used three output files that it generated when running: a *MPlayer output* file, *current time* file and *final output* file.

The role of the *MPlayer output* file was to enable the script to read the current time position in the movie. Every time the BASH script checked for a new TTL pulse (i.e. every 50ms), it would also send a command to *MPlayer* to get the time position in the movie (using the *pausing_keep_force* and *get_time_pos* commands for *MPlayer* in slave mode). As *MPlayer* received commands through a temporary */tmp/doo* file, the script had to pipe the stdout output to the *MPlayer output* file for it to then be able to read the value itself. *MPlayer* only gave the time position up to one decimal. A line inside *MPlayer output* would look like:

ANS_TIME_POSITION=1.6

The script would then read the last line of the *MPlayer output* file and write a new line in the *current time* file. Every line consisted of the newly acquired time position in the movie and a timestamp formed by the Linux epoch time (the number of seconds from 00:00:00 UTC on 1 January 1970) and the milliseconds elapsed since the end of the previous second. A line inside the *current time* file would look like:

1572708345 209 ANS_TIME_POSITION=1.6

If paused, the movie was then rewound by that amount by passing a command to *Mplayer* through ‘slave’ mode. When the scanner was restarted, the movie began within 100 ms of the first TTL pulse (again, because it had to receive at least two pulses). Because of a coding error, version two (v2.1) of the script occasionally fast forwarded when it should have rewound. This affected N = 13 or 15.12% of participants. Because fast forwarding could not be greater than one second and the error affected only 47.44% of the runs for those 13 participants (with the other 52.56% being correctly rewound), data timing quality was not compromised more than the first version of the script on average. After fixing this error, the movies rewound correctly whenever the scanner was stopped for the remaining participants for the remainder of the study (v2.2; N = 44 or 51.16% of participants).

Specifically, whenever the movie was paused or started, the script would write to the *final output*, which would typically contain the following lines:

1567528264 953 start

1567531380 437 pause 1

1567531465 886 rewind −0.592 start

1567534037 162 pause 2

1567534091 303 rewind −0.384 start

1567535208 234 ended

The above example is taken from v2.2 of the script, which included rewind values. To calculate the rewind times, the script would read the last *start* and *pause* lines of the *final output* file:

1567528264 953 start

1567531380 437 pause 1

Because our TR=1s, we started counting the number of total TRs registered from the timestamp of the start value in *final output*. For example, above we would consider 3116 TRs elapsed from 1567528264 953 until 1567531380 953 (1567531380–1567528264). However, as the script stopped the movie at 1567531380 437 only 3115 TRs were registered, meaning that the registered data only went up to 1567531379 953. So, the number of milliseconds of the movie playing without any brain data being acquired would be the difference between 1567531379 953 and 1567531380 437, which would be 437 + 1000 - 953 + 108 = 592. The 108 value was added to account for the fact that it would actually take 108 ms from the moment the script registers the start of a new TR and when the play command is given to *MPlayer*.

The reason behind the coding error in the second version of the script was a minus sign needed when the milliseconds in the pause time were greater than the milliseconds in the start time. The following example is from a correct working version of the script:

1561977334 281 start

1561980159 470 pause 1

1561980228 411 rewind −0.297 start

There would be 2825 TRs registered between 1561977334 281 and 1561980159 281, leaving 470 - 281 = 189 milliseconds lost. The rewind time would be 189 + 108 = 297ms, with a command being sent with a minus sign in front (a lack of a minus sign would fast forward by that amount of ms). To distinguish between the two cases an *if* statement was used. However, in the second version of the script the minus sign was accidentally omitted in one of the branches of the script, resulting in the error described.

Because output files from all versions of the script recorded system and movie timing to calculate start, stop and rewind times, all (including system) delays were tracked and are, therefore, known quantities that can be accounted for in preprocessing to assure that fMRI timeseries and movies are temporally well aligned (see ‘Timing correction’ below).

#### Movie annotations

Words and faces were annotated in the movies using fully automated approaches. These were then used to demonstrate data and timing quality while also illustrating a method for network labelling. For words, we extracted the audio track as a ‘.wav’ and the subtitle track as a ‘.txt’ file from each movie ‘.iso’ file. The.wav file was input into the ‘*Amazon Transcribe*’, a machine learning based speech-to-text transcription tool from *Amazon Web Services* (AWS; https://aws.amazon.com/transcribe/). The resulting transcripts contained on and offset timings for individual words, although not all words are transcribed or accurately transcribed. In contrast, movie subtitles do not have accurate on and offset times for individual words though most words are accurately transcribed. Therefore, to estimate the on and offset times of the words not transcribed, a script was written that first uses dynamic time warping (DTW^74^) to align word onsets from the speech-to-text transcript to corresponding subtitle words in each individual subtitle page, starting 0.5 seconds before and ending 0.5 seconds after the page to account for possible subtitle inaccuracies. In order to improve matches between subtitles and transcripts, punctuation was removed and words stemmed (e.g., ‘kittens’ becomes ‘kitten’). Subtitle words that matched or that were similar to the transcriptions during the DTW procedure inherited the timing of the transcriptions, and were returned to their original unstemmed form. Non-identical words were assigned the word’s transcription timing that had maximum Jaro similarity (given Jaro similarity >0.60) with that subtitle word. Finally, if multiple words in the subtitles aligned with a single transcript word (e.g., ‘is’,‘a’, ‘story’ in the subtitles and ‘story’ in the transcription), we gave the timing of the transcribed word to the matched subtitle or most similar word if the Jaro similarity was >0.60.

Remaining subtitle words not temporally labeled were then estimated, with different degrees of accuracy. Continuous and partial word estimations inherited their on and offset times from matching/similar transcription words in the subtitle page. ‘Continuous’ words use the on and offset times from adjacent words directly, making them the most accurate, e.g., the offset is assigned from the onset of the next matched/similar word. ‘Partial’ estimation occured where there was more than one word between matched/similar words. In those cases the length of each word was approximated, making it less accurate. ‘Full’ estimation was the least accurate, occurring when there were no matching/similar words transcribed, and the onsets and lengths of the words were estimated from the onset and offset of the subtitle page. For partial and full estimations, word length was determined by counting the number of letters in each word and dividing up the bounding time proportionally. For example, if there were two words with 10 and five letters, they got 66.67% and 33.33% of the time, respectively. This procedure might occasionally result in unreasonably long word length estimations. For instance, perhaps because of a long dramatic pause between words, two four letter words in a 10 second window would each be estimated as being five seconds long. In such cases, we used a word truncation algorithm. Specifically, we truncated estimated words <10 letters and more than 2.5 standard deviations from the mean word length in conversational speech (i.e., >1000 ms) to the mean (i.e., 600 ms, based on^75^). As it is common for words more than 10 letters to be longer than 1 second when spoken, estimated word lengths for words with >10 letters and <two seconds were kept. Estimations >two seconds were truncated to 1000 ms. Finally, at the end of these steps, the script did some post-processing. We reordered words based on onset times, removing words with the same timings. If words overlapped, we shifted the start time of the word to the end time of previous words. For numbers (e.g. 32) not correctly identified in the transcription, we changed to the spelled form (‘thirty two’) and re-ran the script.

To obtain machine learning based face annotations, we used the AWS ‘*Amazon Rekognition*’ application programming interface (API) (https://aws.amazon.com/rekognition/). To do this, the original ‘.mpg’ video files were first converted to ‘.mp4’ to have a H264 codec compatible with Amazon’s Rekognition guidelines. A script called the face recognition API without any special configuration or modification and the output was a ‘.json’ file. This contained timestamps every 200 ms, if a face was present, other details about the face (e.g. predicted age range, gender, position on screen and whether the mouth was open) and confidence levels.

### Acquisition

Functional and anatomical images were acquired on a 1.5 T Siemens MAGNETOM Avanto with a 32 channel head coil (Siemens Healthcare, Erlangen, Germany). We used multiband EPI^76,77^ (TR = 1 s, TE = 54.8 ms, flip angle of 75°, 40 interleaved slices, resolution = 3.2 mm isotropic), with 4x multiband factor and no in-plane acceleration; to reduce cross-slice aliasing^78^, the ‘leak block’ option was enabled^79^. Slices were manually obliqued to include the entire brain. A slice or at most a few slices of the most inferior aspect of the cerebellum were occasionally missed in individuals with large heads (see ‘Cerebellar Coverage’ below). This EPI sequence had a software limitation of one hour of consecutive scanning, meaning each movie had at least one break. From 5,470 to 8,882 volumes were collected per participant depending on which movie was watched (Table 2). A 10 min high-resolution T1-weighted MPRAGE anatomical MRI scan followed the functional scans (TR = 2.73 s, TE = 3.57 ms, 176 sagittal slices, resolution = 1.0 mm)^3^.

### Preprocessing

MRI data files were converted from IMA to NIfTI format and preprocessed to demonstrate data quality using mostly the AFNI software suite^80^. Individual AFNI programs are indicated parenthetically in subsequent descriptions.

#### Anatomical

The anatomical/structural MRI scan was corrected for image intensity non-uniformity (‘*3dUniformize*’) and deskulled using *ROBEX*^81^ in all cases except for one participant where ‘*3dSkullStrip*’ performed better. The resulting anatomical image was nonlinearly aligned (using ‘*auto_warp.py*’) to the MNI N27 template brain, an average of 27 anatomical scans from a single participant (‘Colin’)^82^. The anatomical scan was inflated and registered with Freesurfer software using ‘*recon-all*’ and default parameters (version 6.0, http://www.freesurfer.net)^83,84^. Resulting automated anatomical parcellations were used to calculate the extent of cerebellar coverage and to create white matter and ventricle (i.e., cerebral spinal fluid containing) regions of interest that could be used as noise regressors^84^. These regions were resampled into functional dimensions and eroded to assure they did not impinge on grey matter voxels. Finally, anatomical images were ‘defaced’ for anonymity (https://github.com/poldracklab/pydeface).

#### Functional

The fMRI timeseries were corrected for slice-timing differences (‘*3dTshift*’) and despiked (‘*3dDespike*’). Next, volume registration was done by aligning each timepoint to the mean functional image of the centre timeseries (‘*3dvolreg*’). For 23 (or 26.74%) of participants, localiser scans were redone because, e.g., the participant moved during a break and the top slice of the brain was lost. For these participants, we resampled all functional grids to have the same x/y/z extent (‘*3dresample*’) and manually nudged runs to be closer together (to aid in volume registration). For all participants, we then aligned the functional data to the anatomical images (‘*align_epi_anat.py*’). Occasionally, the volume registration and/or this step failed as determined by manual inspection of all data. In those instances we either performed the same procedure as for the re-localised participants (N = 5 or 5.81%) or reran the ‘*align_epi_anat.py*’ script, allowing for greater maximal movement (N = 6 or 6.98%). Finally, the volume-registered and anatomically-aligned functional data were (nonlinearly) aligned to the MNI template brain (‘*3dNwarpApply*’).

Next, we cleaned the timeseries, resulting in what we henceforth refer to as the ‘detrended timeseries’ for each run. Specifically, we first spatially smoothed all timeseries to achieve a level of 6mm full-width half maximum, regardless of the smoothness it had on input (‘*3dBlurToFWHM*’^85^). These were then normalised to have a sum of squares of one and detrended (‘*3dTproject*’) with a set of commonly used regressors^86^: These were (1) Legendre polynomials whose degree varied with run lengths (following a formula of [number of timepoints * TR]/150); (2) Six demeaned motion regressors from the volume registration; (3) A demeaned white matter activity regressor from the averaged timeseries in white matter regions; and (4) A demeaned cerebrospinal fluid regressor from the averaged timeseries activity in ventricular regions.

#### Cerebellar coverage

We quantified the extent of cerebellar coverage in each individual. This was done by extracting the cerebellum from the Freesurfer parcellation (‘*3dROIMaker*’ and ‘*3dcalc*’) and resampling it to functional dimensions (‘*3dresample*’). We made a brain only mask from all runs (‘*3dAutomask*’ and ‘*3dmask_tool*’) and intersected it with the cerebellum. We then fit a box to each of the resulting two images (‘*3dAutobox*’) and calculated the difference in the number of slices in the z-direction.

#### Timing correction

To use stimulus annotations, timing correction was done to account for delays caused by the movie pausing script to assure that fMRI timeseries and movies are well aligned. Specifically, this script introduced a known 100 ms delay that was cumulative for each break in the movie. Furthermore, depending on the versions of the script, there was also a possible additional (cumulative) delay from not rewinding (v1) or occasionally mistakenly fastforwarding (v2.1). These delays were calculated from script output files created for this purpose. Furthermore, the script output files allowed us to quantify potentially variable soft and hardware delays and account for these as well. In particular, every voxel of the detrended timeseries was shifted back in time using interpolation to account for all delays, in the same manner as in slice timing correction but over all voxels uniformly (‘*3dTshift*’).

Specifically, in v1 of the script, if the movie stopped at, e.g., 1000.850 and the last full TR was lost, it means that 850 ms of the movie was watched but is missing from the timeseries. To account for the missing information, we added a TR to the timeseries being collected before the scanner was stopped and interpolated the next run backwards in time the amount not covered by this TR. The added TR was created by retrieving the last timepoint of the run in which the movie was stopped and the first timepoint of the run after the movie was stopped and averaging these. Thus, for the 850 ms of movie watched but dropped, there was 150 ms too much time added to the movie by adding a TR (because our TR = 1 second). Thus, we shifted the next run back this amount so that the timeseries is theoretically continuous again (though this is never really possible). If there was another run (i.e., three or more), the same logic applied except that the extra 150 ms needed to be accounted for. So, if the next run stopped at 2000.900, we shifted run three back (1000-900) + 150 ms = 250 ms.

These calculations are complicated by the fact that each scanner stop always creates a 100 ms delay and a known standard deviation, because of the way the *MPlayer* script works (see ‘Movie Pausing’). For this reason, every run is time shifted backward this extra amount. So in the example, if this delay was 100 ms, run three in the prior example would be shifted back 350 ms. Version 2.2 of the script is simpler: an additional TR is not added and the only time shifting corresponds to the time lost whenever the scanner was stopped from monitoring for the TTL pulse. For example, if there are three runs and 100 ms was lost each run, the final run would be time shifted back 300 ms. That is, the cumulative delay is the only time shifting done. In the v2.1 script, the timing correction was carried out as in the prior paragraph to account for a coding error when it occurred or as in v2.2 when it did not.

#### ICA artifact removal

Spatial independent component analysis (ICA) is a powerful tool for detecting and removing artifacts that substantially improves signal-to-noise ratio in movie naturalistic-fMRI data^87^. First, we concatenated all detrended timeseries after timing correction. As in the HCP, we did spatial ICA on this timeseries with 250 dimensions using ‘*melodic*’ (version 3.14) from FSL^88^. Next, we labelled and removed artifacts from timeseries, following an existing guide for manual classification^89^. One of three trained authors went through all 250 components and associated timecourses, labelling the components as ‘good’, ‘maybe’, or ‘artifact’. As described in Griffanti *et al*.^89^, there are a typical set of ‘artifact’ components with identifiable topologies that can be categorised as ‘motion’, ‘veins’, ‘arteries’, ‘cerebrospinal fluid pulsation’, ‘fluctuations in subependymal and transmedullary veins’ (i.e., ‘white matter’), ‘susceptibility artefacts’, ‘multi-band acceleration’ and ‘MRI-related’ artefacts. Our strategy was to preserve signal by not removing components classified as ‘maybe’. On a subset of 50 datasets (58.14% of the data), a second author classified all components to check for consistency. The authors discussed discrepancies and modified labels as warranted. It was expected that, similar to prior studies, about 70–90% of the 250 components would be classified as artifacts^89^. Once done, we regressed the ICA artifact component timecources out of the detrended and concatenated timeseries (‘*3dTproject*’).

### Analyses

We used the preprocessed, detrended and concatenated timeseries with ICA-based artifacts removed (henceforth ‘fully detrended timeseries’) for several analyses meant to validate data quality. These included calculating the temporal signal-to-noise (tSNR) ratio as one of a set of metrics and a composite measure to assess data quality at the timeseries level (Overall Data Quality). We also did two whole-brain functional analyses using two previously established data-driven methods. One was intersubject correlation (ISC) analysis and the other involved labelling functional networks with annotations (Network labelling). These serve to show data quality similar to past work and provide evidence for timing accuracy between fMRI timeseries for participants and movies. The latter is crucial as movie breaks varied across participants, resulting in a small amount of temporal interpolation and psychological discontinuity across runs.

#### Temporal signal-to-noise ratio

We calculated tSNR both before and after minimal preprocessing to demonstrate data quality. We also calculated tSNR after extensive preprocessing to show how it might improve after timeseries cleaning and artifact removal (though it will generally increase with increasing signal removal). Temporal SNR can be defined as the mean signal divided by the standard deviation of the signal over voxel timeseries^90^. Though multiband acceleration greater than one improves sensitivity over multiband one^78^, average multiband four tSNR tends to be between 40–60, lower than unaccelerated sequences^78,88^. A movie naturalistic-fMRI dataset showed that manual ICA-based artifact rejection increased tSNR around 50 units, though this was not multiband data^87^. HCP multiband four tSNR increased by 30 after ICA cleanup of resting-state data^88^. Thus, we would expect to see a similar baseline level and improvement after ICA artifact removal. It is worth noting that unlike most other datasets, we have over 1.5 hours of data per participant, likely sufficient at those tSNR values for detecting effects sizes of 1% or less^91^.

We first calculated tSNR (‘*3dTstat*’) on three timeseries: 1) A minimally preprocessed timeseries that was corrected for slice timing, despiked, volume-registered and aligned to the anatomical image, timing-corrected and concatenated; 2) The same timeseries but blurred with a 6 mm FWHM (‘*3dBlurToFWHM*’); and 3) A fully preprocessed timeseries, detrended using white matter, ventricular, motion and ICA artifact timecourse regressors (‘*3dTproject*’). We then calculated mean tSNR for all three timeseries using a mask that included grey matter, with most white matter and ventricle voxels removed. We calculated effect sizes at a voxel level using:

$$Cohen{\prime} s\,d=\frac{{M}_{1}-{M}_{2}}{\sqrt{\frac{(S{D}_{1}^{2}-S{D}_{2}^{2})}{2}}}$$

where *M* and *SD* are the mean and standard deviation of the tSNR in a voxel for the more (subscript one) minus the less preprocessed timeseries (subscript two). Thus, a positive Cohen’s d represents larger tSNR for more preprocessed timeseries.

#### Overall data quality

Timeseries data quality were globally assessed using 10 measures and a composite of these: 1) To quantify timeseries timepoints outliers, we labelled voxels that were far from the median absolute deviation (‘*3dToutcount*’). Whole timepoints were defined as outliers if more than 10% of voxels were outliers by this definition; 2–8) We used seven parameters to quantify motion. These included the maximum average motion for each run from the demeaned motion regressors and the largest change in displacement between two successive timepoints (Delta); 9) The mean tSNR from the minimally preprocessed timeseries; and 10) The total number of ‘artifact’ ICA components. We then used the ‘*multicon*’ package in R (https://www.r-project.org/) to z-transform these 10 items and create a composite data quality score for each participant. We defined outlying participants as anyone whose composite score was more than three standard deviations from the mean. In addition to these measures, we also ran MRIQC (version 0.15.2; https://github.com/poldracklab/mriqc)^92^, a tool for automated extraction of no-reference image quality metrics. MRIQC results might allow for a more systematic comparison of our data with other datasets given its increasing use (e.g., using https://github.com/elizabethbeard/mriqception).

#### Intersubject correlation

In addition to illustrating data quality similar to prior results, ISC demonstrates synchrony of fMRI timeseries between our participants. This would presumably not occur unless the movies were accurately aligned after timing correction. We compared the ISCs of participants watching the same movie to those watching different movies because a fundamental assumption of ISC is that synchrony is stimulus driven. Thus, we expected correlation values to be significantly greater for the same movie compared to different movies, with values similar to past ISC results from a large number of participants. For example, in a task-fMRI study with 130 participants, the maximum ISC is 0.27^93^.

Because movies had different lengths, we first truncated the fully detrended timeseries to be the length of the movie with the shortest duration (i.e., ‘*500 Days of Summer*’; 5470 s/TRs or about 1 hour and 31 minutes). We then computed pairwise Pearson’s correlations between the timeseries in each voxel for all pairs of participants for all movies (‘*3dTcorrelate*’). This resulted in (1/2 * 86 * (86-1)) = 3655 pairwise correlation maps. These are composed of (1/2 * 20 * (20-1)) + (1/2 * 18 * (18-1)) + ((1/2 * 6 * (6-1)) * 8) = 463 maps from participants watching the same movie. The remaining (3655 - 463) = 3192 maps are from participants watching different movies.

For the group analysis, we first converted Pearson’s r values to be normally distributed as z-scores using the Fisher z-transformation. Then, to compare ISC maps from people watching the same or different movies, we used voxel-wise linear mixed effects models with crossed random effects (‘*3dISC*’). This approach accounts for the interrelatedness of the pairwise ISC maps and can handle unequal sample sizes^94^. The resulting map was Bonferroni-corrected for multiple comparisons using *t* = 6.04 corresponding to a voxel-wise p-value of 0.01 divided by the number of tests done in each voxel, i.e., *p* < 0.01/(4 * 64,542) = 0.00000004. We combined this with an arbitrary cluster size threshold of 20 voxels. To demonstrate reliability, we also repeated this analysis after splitting the data into groups of participants watching two different sets of five movies. We compared the resulting spatial patterns of activity using two standard approaches, correlation and eta^2,95^ (‘*3ddot*’).

#### Network labelling

Besides demonstrating data and timing quality, here we also illustrate a fairly straightforward method for using annotations to label networks with a method similar to one used in existing naturalistic-fMRI studies. This combines model-free ICA to find networks and a model-based approach to label those networks^96,97^. In particular, we derive networks in each participant with ICA using ‘*melodic*’ run on the fully detrended timeseries (and, again, limited to 250 dimensions). We then convolve annotated word, no word, face and no face onsets and durations with a canonical hemodynamic response function (‘*3dDeconvolve*’). The resulting ideal waveforms are regressed against the 250 independent component timescourses using general linear model (GLMs) followed by pairwise contrasts between words and no words and faces and no faces (using FSL’s ‘*fsl_glm*’). A Bonferroni-corrected threshold was set at p = 0.01 at the single voxel level divided by 250 components and eight statistical tests (not all of which are discussed here), i.e., 0.01/(250 * 8) = p < 0.000005. We combined this with an arbitrary cluster size threshold of 20 voxels at the component level. If there was more than one resulting component at this threshold and cluster size, we summed those components.

For group analysis, we did one sample t-tests for GLM results of words vs no words, no words vs words, face vs no faces and no faces vs faces (‘*3dttest*++’). To correct for multiple comparisons, we again used a Bonferroni correction of 0.01 at the single voxel level divided by approximately 85,000 voxels and four tests, i.e., 0.01/(85,000 * 4), rounding to p < 0.00000001. We again combined this with an arbitrary cluster size threshold of 20 voxels. To illustrate the precise anatomical correspondence of our results with prior data, we overlay fMRI term-based meta-analysis from Neurosynth^98^ (Retrieved May 2020) for ‘language’ (https://neurosynth.org/analyses/terms/language/; from 1101 studies) and the ‘fusiform face’ area (https://neurosynth.org/analyses/terms/fusiform%20face/; from 143 studies; FFA). We further illustrate anatomical correspondence by showing the mean peaks of the putative (left and right) FFA, derived by averaging peaks from a meta-analysis of 49 studies (converted to MNI x/y/x coordinates = 39/−53/−22 and −40/−54/−23; see Table 1 in^99^).

## Data Records

Information and anatomical data that could be used to identify participants has been removed from all records. Resulting files are available from the OpenNeuro platform for sharing fMRI (and other neuroimaging) data at https://doi.org/10.18112/openneuro.ds002837.v1.1.1 (dataset accession number ds002837)^100^. A README file there provides a description of the available content. The code/scripts used for this manuscript are available on GitHub (https://github.com/lab-lab/nndb; Online-only Table 1). Additional material will also be made available on the NNDb website (http://www.naturalistic-neuroimaging-database.org).

### Participant responses

**Location** nih_demographics.csv, nih_data.csv and nih_scores.csv

**File format** comma-separated value

Participants’ responses to demographic questions and the NIH Toolbox in comma-separated value (CSV) files. Data is structured as one line per participant with all questions and test items as columns.

### Anatomical MRI

**Location** sub-<ID>/anat/sub-<ID>_T1w.nii.gz

**File format** NIfTI, gzip-compressed

**Sequence protocol** sub-<ID>/anat/sub-<ID>_T1w.json

The defaced raw high-resolution anatomical images are available as a 3D image file, stored as sub-<ID>_T1w.nii.gz.

The N27 MNI template aligned anatomical image and the anatomical mask with white matter and ventricles removed are also available as derivatives/sub<ID>/anat/sub-<ID>_T1w_MNIalignment.nii.gz and derivatives/sub<ID>/anat/sub-<ID>_T1w_mask.nii.gz respectively

### Functional MRI

**Location** sub-<ID>/func/sub-<ID>_task-[movie]_run-0[1–6]_bold.nii.gz

**Task-Name [movie]** 500daysofsummer, citizenfour, theusualsuspects, pulpfiction, theshawshankredemption, theprestige, backtothefuture, split, littlemisssunshine, 12yearsaslave

**File format** NIfTI, gzip-compressed

**Sequence protocol** sub-<ID>/func/sub-<ID>_task-[movie]_run-0[1–6]_bold.json

The fMRI data are available as individual timeseries files, stored as sub-<ID>_task-[movie]_run-0[1–6]_bold.nii.gz. The fully detrended timeseries is also available as derivatives/sub-<ID>_task-[movie]_bold_preprocessedICA.nii.gz.

### Motion and outlier estimates

**Location** derivatives/sub-<ID>/motion/sub-<ID>_task-[movie]_run0[1-6]_bold_[estimates]0.1D

**Motion [estimates]** motion, maxdisp_delt, wm, ventricle and outliers

**File format** plain text

Motion estimates are from the registration procedure in the AFNI program ‘*3dvolreg*’ and outliers were estimated using ‘*3dToutcount*’, These are provided in space-delimited text files where the estimates represent 1) *motion*: degree of roll, pitch and yaw and displacement in the superior (dS), left (dL) and posterior (dP) directions in mm; 2) *maxdisp_delt*: maximum displacement (delta) between any two consecutive timepoints; 3) *wm*: mean activity in the white matter; 4) *ventricle*: mean activity in the ventricles and 5) *outliers*: individual timepoint outliers at 10% levels.

### ICA artifact labels

**Location** derivatives/sub<ID>/func/sub-<ID>_task-[movie]_bold_ICAartifacts.1D

**File format** plain text

ICA components labeled as artifacts used to correct ICA timeseries as proved are provided as space delimited text where the columns are artifactual timecources.

### MRIQC

**Location** derivatives/mriqc

**File format** plain text/html/json

This contains a large collection of image quality metrics. A complete description of these can be found at https://mriqc.readthedocs.io/en/stable/measures.html.

### Annotations

**Location** stimuli/task-[movie]_[annot]-annotation.1D

**Annotation [annot]** word, face

**File format** plain text

Word, no word and face and no face onsets and durations are provided in four space-delimited text files. In the word annotation file, columns represent: 1) Words; 2) Word onset in seconds and milliseconds; 3) Word offset in seconds and milliseconds. In the face annotation file, columns represent: 1) Face onset in seconds and milliseconds and 2) Duration of face presence in seconds and milliseconds.

## Technical Validation

### Stimuli

#### System timing

The movies were played in the original DVD audio and video quality. This relative lack of compression results in low latencies when starting and stopping the movies. System delays were calculated from the timing output of the movie-pausing script. Averaged over all runs and participants, this delay was 19.73 ms (SD = 7.57). This is perhaps not more than the expected latency on a standard Linux kernel^101^. However, because this delay was measured, it can be accounted for in the timeseries through temporal interpolation as described.

#### Annotations

Words and faces were annotated in the movies so that they could be used to show data quality and timing accuracy while also illustrating a fairly straightforward method to label brain networks. To be used for this purpose, the overall quality of the annotations themselves needs to be demonstrated. For words, Table 3 provides a breakdown that reflects relative word on and offset accuracy for individual movies. Machine learning-based speech-to-text word transcriptions are assumed to have the highest temporal accuracy. An average of 75.75% of subtitle words had matching or similarity-matched word transcriptions. This was after hand transcribing over 2000 missing word times for ‘*Little Miss Sunshine*’ to bring accuracy up to 72.48% in order to correct for poor transcription accuracy (~45%, possibly due to overlapping dialogue in the movie). Speech-to-text transcription left an average of 24.25% of the subtitle words to get estimated word lengths. Of these, an average of 20.30% were made up of the ‘continuous’ and ‘partial’ estimations, considered relatively accurate because they rely on accurately transcribed matched/similar words to make estimations. Only 3.95% of the subtitle words on average were fully estimated. These have the least accurate word timings because their length had to be estimated entirely from the subtitles page start and end times. Finally, to increase accuracy we truncated the 2.52% of words that were unreasonably long. In summary, it might be argued that about (75.75% Matched/Similar + 20.30% Continuous/Partial) = ~95% of words have relatively accurate millisecond level onset times. Given that there are >10,000 words on average per movie, a ~5% rate for less accurate word timing is likely acceptable.

**Table 3:** Movie word and face annotation information.

Movie	Words						Faces	
	On and Offsets (%)				Truncated	N		
	Matched/Similar	Estimated						
		Continuous	Partial	Full			>95% (%)	Time (%)
500 Days of Summer	65.46	4.57	21.84	8.12	4.13	8,286.00	93.15	80.83
Citizenfour	80.68	3.82	14.62	0.88	1.32	13,936.00	93.04	70.79
12 Years a Slave	67.41	6.06	19.66	6.86	3.64	7,984.00	88.48	77.54
Back to the Future	72.52	4.40	17.32	5.77	2.35	8,634.00	89.85	71.21
Little Miss Sunshine	72.48	3.18	22.47	1.87	3.12	8,555.00	87.96	79.17
The Prestige	77.22	4.69	15.19	2.89	2.39	10,954.00	88.84	77.09
Pulp Fiction	73.14	4.13	18.35	4.38	2.77	16,155.00	88.88	79.63
The Shawshank Redemption	81.62	4.92	10.86	2.60	2.12	11,779.00	85.30	78.55
Split	82.21	4.34	8.58	4.88	2.09	7,032.00	96.27	70.13
The Usual Suspects	84.80	3.36	10.61	1.23	1.27	9,913.00	94.94	74.12
Mean	75.75	4.35	15.95	3.95	2.52	10,322.80	90.67	75.91
SD	6.57	0.82	4.83	2.46	0.92	2,909.40	3.49	4.01

The on and offsets of words were obtained from machine learning-based speech-to-text transcriptions. Dynamic time warping was used to align these to subtitles. If words in a subtitle page ‘Matched’ or were ‘Similar’ to words in the transcript, it received the transcript timing. Otherwise it was estimated. ‘Continuous’ estimations are single subtitle words inheriting the start and end time from the end of the prior and start of the next transcribed word. ‘Partial’ estimations are similar but involve two or more missing words between transcribed words. ‘Full’ estimations occured when no words were transcribed and words were estimated from the start and end time of the subtitle page. When word lengths were unreasonable, they were ‘Truncated’. This procedure resulted in an average number (‘N’) of >10,000 words per movie. The on and offsets of faces were also obtained from a machine learning-based approach. The final two columns are the average percentage of face labels with >95% confidence and the percent of time faces were on screen.

For face labels, histograms for all movies were used to examine the distribution of confidence levels. Across all movies, the average percentage of face labels with a confidence value greater than 0.95 was 90.67%, motivating us to use all the labelled faces in further analysis (Table 3). We also qualitatively compared results with the movies and they appeared to confirm that confidence levels were accurate.

### Anatomical MRI

Table 4 provides a list of anatomical and fMRI irregularities. Anatomical image segmentation and cortical surface reconstruction with Freesurfer finished without error for all participants. Surfaces were individually inspected and no manual corrections were needed, suggesting anatomical images were of good quality.

**Table 4:** Data acquisition irregularities that might have impacted data quality.

BIDS ID	Movie	Irregularity	
		Hardware/Scanning	Participant
1	500 Days of Summer	Anatomical scan first	Paused to adjust volume
14	500 Days of Summer	30 channel headcoil	
16	500 Days of Summer	Given glasses at first break	Paused once b/c drowsy
21	Citizenfour		Paused once b/c drowsy
24	Citizenfour	Anatomical different day	
25	Citizenfour		Appeared drowsy
28	Citizenfour		Paused b/c fell asleep once
33	Citizenfour		Appeared drowsy
35	Citizenfour		Appeared drowsy
37	Citizenfour		Paused once b/c drowsy
38	Citizenfour		Paused once b/c drowsy
47	Pulp Fiction	Paused to adjust earphones	
51	The Shawshank Redemption	Wrong EPI sequence (2.8 s)	
55	The Shawshank Redemption	Paused to clean glasses	
59	The Prestige		Paused once b/c drowsy
63	Back to the Future		Appeared drowsy
65	Back to the Future		Paused to adjust volume
73	Split	Arduino issue/extra pauses	
80	Little Miss Sunshine		Paused once b/c drowsy

Most irregularities centred around participant drowsiness. We monitored participants through a camera and occasionally gave them warnings if they appeared drowsy to us. In a few cases we paused the scan to let participants compose themselves and to make sure they would remain alert throughout the rest of the scan.

### Functional MRI

#### Cerebellar coverage

Overall, most of the cerebellum was scanned for all participants. Specifically, 68.60% of participants were missing zero slices (34.88%) or one slice (33.72%) of the cerebellum. The mean number of missing cerebellar slices across all participants was 1.19 (or 3.19 mm; SD = 1.22 slices; Maximum = 5 slices).

#### Movie pausing

To maintain alignment between the movies and fMRI timeseries, movies were played with as few breaks as possible. In 93.94% of cases, the experimenter paused the scans every 41.54 minutes on average (SD = 10.47). Only ten participants initiated pauses in 16 runs or 6.06% of all runs (Table 4). Thus, there was a relatively low number of runs (M = 3.07; SD = 0.98) despite that movies were up to two hours and 28 minutes long.

The timing for one dataset needed additional corrected due to technical issues with the Arduino device wires on the day of the scan (Table 4). The Arduino mistakenly stopped transmitting the TTL pulse, likely because of a loose wire, registered by the BASH script as pauses when the scan was still ongoing. Thus, instead of the two actual pauses, eight were recorded, meaning six of the alleged pauses did not occur. The false pauses added eight seconds to the timing output file as the scan was still ongoing, increasing the apparent total length of the movie by 48 seconds, and therefore increasing scan time as a consequence. In order to correct for this error, eight TRs were removed from the timeseries whenever a false pause was detected, for a total of 48 TRs removed.

#### Temporal signal-to-noise ratio

**Fig. 2:**
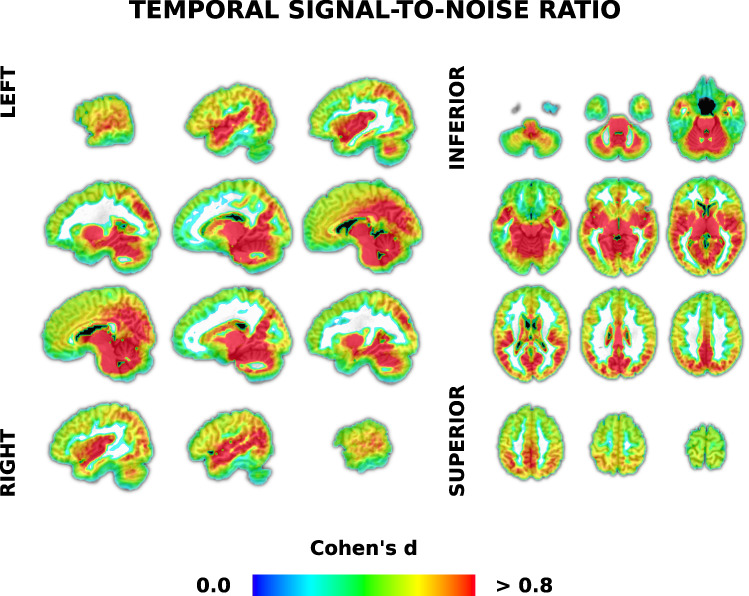
Voxel-wise temporal signal-to-noise ratio analysis demonstrating increases in data quality with preprocessing. Temporal SNR was calculated in each voxel using mostly unprocessed and fully preprocessed functional magnetic resonance imaging (fMRI) timeseries data from 86 participants. Full preprocessing included blurring and detrending using motion, white matter, cerebral spinal fluid and independent component analysis (ICA) based artifact regressors. Cohen’s d effect sizes were calculated in each voxel as the mean differences between fully preprocessed and minimally preprocessed fMRI timeseries tSNR, divided by the pooled standard deviation. See Table 5 for tSNR values averaged across grey matter voxels.

Mean tSNR for timeseries averaged over grey matter voxels was comparable to prior multiband four studies reviewed earlier. Furthermore, there were comparable increases in tSNR after preprocessing (Table 5). Cohen’s d at the individual voxel level shows regions of the brain for which tSNR increased after full preprocessing (Fig. 2). This includes most medial and posterior aspects of the brain, with less tSNR increase in the frontal lobe.

**Table 5:** Descriptive statistics of temporal signal-to-noise ratio and independent component analysis based measures of data quality across movies.

Measure	tSNR						
	Mean			Maximum			ICA artifacts (%)
	Min Pre	Blur Pre	Full Pre	Min Pre	Blur Pre	Full Pre	
Min	11.85	12.20	13.37	91.33	110.33	185.85	56.40
Mean	39.43	44.55	63.82	161.19	201.20	319.18	71.71
SD	10.17	12.73	20.79	29.15	40.16	50.58	7.47
Max	60.10	68.99	98.03	218.09	310.91	431.79	87.20

The temporal signal-to-noise ratio (tSNR) was calculated in mostly grey matter for minimally preprocessed (‘Min Pre’), blurred (‘Blur Pre’) and fully detrended and preprocessed (‘Full Pre’) functional magnetic resonance imaging timeseries data (see also Fig. 2). The final column is the percent of manually-labelled independent component analysis (ICA) artifacts out of 250 dimensions.

#### Overall data quality

We assessed overall fMRI timeseries data quality using 10 measures and a composite of these. Table 6 shows the means per run across participants for eight of these measures. With the exception of run three, the number of outlying timepoints was under 1% per run on average. Maximum motion, as measured by six motion regressors, was low, under a degree and millimeter on average. The greatest maximal displacement was pitch (0.96°) and movement in the inferior/superior direction (0.92 mm). This is perhaps what might be expected for supine participants whose heads are firmly held in the left/right directions. Maximum delta was similarly under one millimeter. These parameters did not increase more in later compared to earlier runs. If anything, maximal movement decreased over the scanning session.

**Table 6:** Descriptive statistics for outlying timepoints, motion and timing measures of data quality averaged over movie runs.

Break	N (%)	Time (%)	Outliers (%)	Maximum Motion (° or mm)							
				Roll	Pitch	Yaw	I/S	L/R	A/P	Delta	Interp (-ms)
1	100.00	40.34	0.19	0.51	1.01	0.54	1.04	0.40	0.60	0.82	123.97
2	100.00	34.28	0.18	0.47	0.91	0.55	0.84	0.38	0.52	0.83	422.23
3	69.77	17.81	1.59	0.45	0.99	0.46	0.81	0.34	0.53	0.85	675.70
4	26.74	6.15	0.15	0.48	0.92	0.54	0.95	0.44	0.56	0.94	747.96
5	6.98	0.91	0.04	0.36	0.57	0.35	0.46	0.20	0.42	0.64	1,171.83
6	3.49	0.50	0.00	0.29	0.38	0.25	0.33	0.18	0.16	0.28	1,168.33
		wMean	0.43	0.48	0.96	0.53	0.92	0.38	0.55	0.83	377.68
		wSD	0.47	0.03	0.06	0.04	0.10	0.03	0.04	0.05	214.00

‘N’ the percentage of 86 participants having up to six breaks during any given movie. ‘Time’ is the average percentage of the whole movie for the run preceding each break. ‘Outliers’ is the mean percentage of timepoints with greater than 10% outliers in each run. Motion includes the mean maximum deflection in the inferior/superior (‘I/S’), left/right (‘L/R’) and anterior/posterior (‘A/P’) directions and the mean maximum change between any two timepoints (‘Delta’) in millimeters (mm; see main text for Frame Displacement). ‘Interp’ is the amount timeseries were interpolated back in time in milliseconds (-ms) in each run on average to account for known delays. The bottom two rows are the weighted means (wMean) and standard deviations (wSD) of rows weighted by the Time column.

The two other measures of overall data quality are given in Table 5, showing that tSNR (discussed in the prior section) and the number of ICA artifacts were reasonably high and low, respectively. On a subset of 50 datasets, there was a 96.22% agreement (SD = 2.20) between authors with regard to ICA artifact classification. Percentages of ICA artifacts are similar to those found in prior studies reviewed earlier. Finally, we created a composite measure from these 10 metrics to detect outliers (reverse coding tSNR). These measures had a high internal consistency with Cronbach’s alpha = 0.94. Using this measure, only one participant was considered an outlier. This is the participant mentioned in the Methods/Participants that was excluded from the database. Taken together, these measures indicate that NNDb timeseries data are of high overall quality.

Results from MRIQC were largely consistent with the above, demonstrating that the data is of high quality, similar to or better than other datasets using this tool^102–105^ (see also https://github.com/elizabethbeard/mriqception). To give two examples: Mean tSNR was 38.43 (SD = 7.540), nearly identical to our own calculation. Framewise Displacement (FD) is an increasingly standard method for calculating instantaneous head-motion^106^. The mean FD was 0.138 mm (SD = 0.06) and there were 34.52 (SD = 74.72) timepoints above a 0.50 mm threshold on average (of 6998.20 timepoints or 0.49% of the data on average). Our mean FD compares favorably to 0.12^107^, 0.13^108^, 0.15^109^, 0.18^110^ and 0.24 mm^111^ in other studies with typically developing participants watching videos or movies during fMRI.

#### Intersubject correlation

**Fig. 3:**
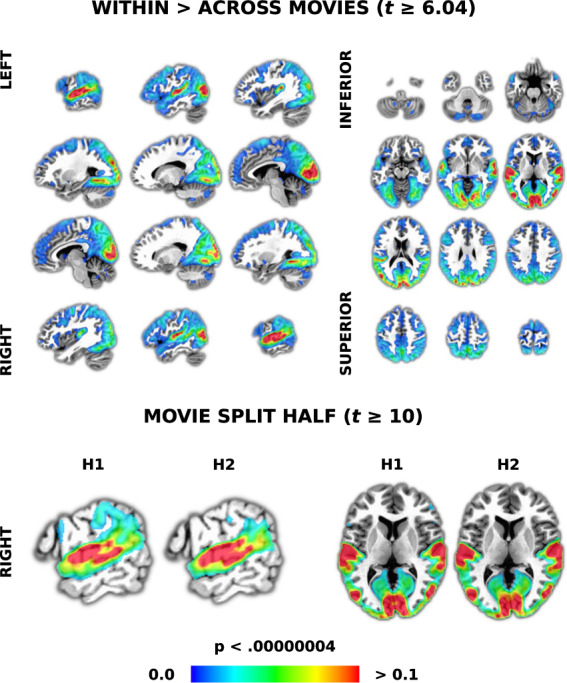
Results of intersubject correlation (ISC) demonstrating data quality and timing synchrony between participants and movies. ISC is a data-driven approach that starts with calculating the pairwise correlations between all voxels in each pair of participants. We used a linear mixed effects with crossed random effects (LME-CRE) model to contrast participants watching the same versus different movies (top). Equally-spaced slices were chosen to be representative of results across the whole brain. To demonstrate reliability, we split the data in half, with each having five different movies. The same LME-CRE model was run on each half and the results are presented at an arbitrary threshold to more easily view similarities and differences (bottom row). Slices were chosen to make differences more salient. The colour bar represents correlation values (*r*) in all panels. All results are presented at a p-value corrected for multiple comparisons using a Bonferroni correction and an arbitrary minimum cluster size threshold of 20 voxels.

ISC was undertaken to show functional fMRI data quality and timing accuracy by demonstrating synchronization between participants and movies. There was significantly higher ISC at a Bonferroni-corrected threshold in large portions of auditory and visual cortices (precisely following sulci and gyri) when participants watched the same movies compared to different movies (Fig. 3, top). Similar to prior work, the maximum correlation was *r* = 0.28. To examine reliability, we split the movies into two groups of participants that watched different sets of five movies. The results were largely spatially indistinguishable from each other (*r* = 0.96; eta^2^ = 0.98) or from results with all movies (with *r/*eta^2^ of 0.991/0.995 and 0.988/0.994). Even when the data was thresholded at a *t*-value of 10, an arbitrary value chosen because even extremely high p-values resulted in whole-brain ISC, the results were spatially similar (*r* = 0.82; eta^2^ = 0.91; Fig. 3, bottom). These results demonstrate high data quality through robust activity patterns, spatial precision and timing accuracy through participant synchrony with movies.

#### Network labelling

**Fig. 4:**
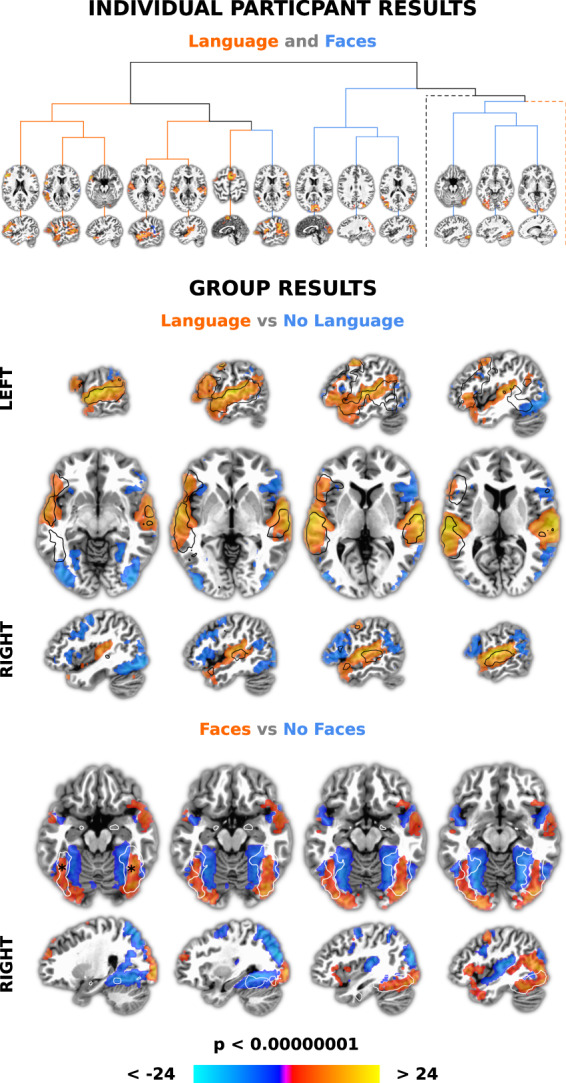
Results of combined independent component analysis (ICA) and model-based analysis demonstrating data quality, timing accuracy and an approach to network labelling. First, networks were found at the individual participant level using ICA, a multivariate data-driven approach. Word and face annotations from movies were then convolved with a standard hemodynamic response function and used in general linear models to find associated IC timecourses. The dendrogram (top) shows 13 of 20 significant networks from an example participant that were more associated with words > no words (‘Language’; red lines) and faces > no faces (‘Faces’; blue lines), clustered to show IC timecourse similarity. Slices are centred around the centre of mass of the largest cluster in each network. Two branches (dotted lines) were excluded for visibility. These had an additional five language and two face networks. For group analysis, spatial components corresponding to significant IC timecourses for each participant were summed and entered into *t*-tests. The middle panel shows that word > no word networks (‘Language’; reds) overlap a ‘language’ meta-analysis (black outline) more than no word > word networks (‘No Language’; blues). Slices are centred around the centres of mass of the two largest clusters, in the left and right superior temporal plane. The bottom panel shows that face > no face networks (‘Faces’; reds) produced greater activity than no face > face networks (‘No Faces’; blues) in the same areas as a ‘fusiform face’ area (FFA) meta-analysis (white outline). Slices are centred near the average x/y/z coordinates of the putative left and right FFA (indicated with black asterisks). The colour bar represents z-scores in all panels. All individual and group level results were Bonferroni corrected for multiple comparisons and presented with an arbitrary minimum cluster size of 20 voxels.

ICA and regression with a canonical response function were used to demonstrate data quality, timing accuracy and an approach to network labelling. There were M = 11.43 (SD = 4.31) word > no word, M = 13.52 (SD = 7.21) no word > word, M = 8.71 SD = 8.09, face > no face and M = 8.44 (SD = 7.84) no face > face networks per participant, each significant at a stringent Bonferroni-corrected threshold. For words (compared to no words), these networks variously consisted of activity in the superior temporal plane, posterior inferior frontal gyrus and motor regions as might be expected during language processing^112^. For faces (compared to no faces), activity was in the posterior superior temporal sulcus and fusiform gyrus among other regions that might be expected during face processing. An example from a single participant is shown in Fig. 4 (top), using hierarchical clustering (with Ward’s method) to order all significant word > no word and face > no face networks in terms of the Euclidean distance between IC timecources to show network similarity. By this approach language and face networks mostly cluster separately.

Group t-tests across participants showed significant patterns of activity consistent with the sum of networks from individual participants, again using a Bonferroni corrected threshold. In particular, word and face networks resembled meta-analyses of language (Fig. 4, middle, black outline) and face processing (Fig. 4, bottom, white outline). The face networks included the putative fusiform face area(s) (Fig. 4, bottom, black asterisks), with immediately adjacent regions more involved in processing times in the movie when faces are not visible. Overall, both individual and group results demonstrate high data quality by being robust and showing anatomical precision. Furthermore, such strong relationships between stimulus annotations and idealised timeseries again indicate that timing accuracy is high.

## Usage Notes

We think that the NNDb has the potential to help revolutionise our understanding of the complex network organization of the human brain as it functions in the real-world. However, there are several limitations and usage bottlenecks, including annotations and analyses that we now discuss to help others use the NNDb to make new discoveries. We conclude by briefly discussing the future expansion of the NNDb.

### Limitations

First, with respect to data acquisition, the study was conducted at 1.5 T. Had it been conducted at 3 T, SNR would theoretically double. However, in practice SNR is only about 25% better and susceptibility artefacts are worse at 3 T^113^. That said, future versions of the database will also include 3 T movie data. Second, because of pausing during acquisition and associated (but known) delays, the fMRI timeseries require temporal interpolation to align to the movies. Thus, anyone using the dataset and not using derivatives, will need to make these corrections. We provide the times and an implementation of this in the AFNI environment (see Online-only Table 1 for the location of the code).

With regard to stimuli, it should be acknowledged that neither the fMRI setting nor movies themselves are necessarily ‘natural’ or realistic^114,115^, though they are certainly more ‘naturalistic’ on the continuum defined in the ‘Naturalistic fMRI’ section. In addition to the obvious fact that participants are in a magnet, there is continual rhythmic noise. We did not use noise cancelling headphones though the inserts were noise attenuating and we further insulated the participants from noise (and movement) with pillows covering their ears. All participants said that they heard the movies well.

There are a few other general issues with using movie stimuli. First, we cannot publicly release the stimuli themselves because of copyright restrictions. As such, we have provided links so that researchers can purchase the same version of the movies (e.g., to make annotations, discussed next). Second, movies are long. Though this does not seem to adversely affect motion, it could be problematic for some (e.g., clinical) populations in future work. Furthermore, for clinical ‘biomarker’ purposes^71,72^, long movies might be too expensive even if patients could sit still for 1.5 hours or more. However, there is no a priori reason that models cannot be trained on (e.g., network based representations of) NNDb data but tested on shorter purpose built subsets of movies.

Finally, there are a few limitations with regard to the participants and functional network analysis we did. First, the 10 participants asking for breaks might have a different pattern of activity before breaks. However, if it is assumed that this lasts for 20 seconds, it means that only 0.06% of the data were affected. This is unlikely to have a big impact on the results. Indeed, we censored timepoints during that time in five participants and it made no discernible difference to results. Second, movies obviously vary on a number of high (e.g., direction style) and low level properties (e.g., brightness). Having 10 different films means that words/faces appeared in a high variety of contexts, likely meaning the results are less confounded with these properties. Nonetheless, we did not control them (beyond including ‘Movie’ as a factor in the ISC models) because they were not yet annotated. These ‘controls’ and other annotations will need to be made but, as we next discuss, this is a potential bottleneck to usage.

### Annotation bottleneck

In many usage cases, annotations will likely be necessary for testing hypotheses with the NNDb. This involves not only coding a stimulus feature of interest but also a suitable range of controls at a finer level of detail than used to label ‘language’ and ‘face’ networks herein. For example, if one were interested in the neurobiological mechanisms of how observed face movements are used by the brain during speech perception^116,117^, one might want to annotate a large range of features. These might include speech with more or less environmental noise when the face is visible (as audiovisual speech improves speech in noise). There might need to be annotated auditory-only controls matched for auditory/semantic features and visible scene complexity. There may need to be face-only controls or audiovisual controls with faces in profile, etc. If done manually, movie annotations at this level of detail will be very time consuming. Though this might prove necessary for testing some hypotheses, we suggest automated approaches and a brain-driven approach that might be used to speed up the annotation process.

Automated approaches to annotation can make use of a large number of existing text-based descriptions of movies to provide time-locked features. These include, (1) Detailed descriptions in scripts that can be aligned to movie times from subtitles^118^; (2) Detailed verbal descriptions from descriptive video services that make movies accessible to millions of visually impaired and blind individuals^119^; (3) Video clips from movies available on social websites like YouTube that can be matched to movie times by visual scene matching to include user comments as features^120^. For example, one two-minute clip from Gravity on YouTube currently contains over 3,800 comments that can be text-mined for features; and (4) There are many emerging automated machine learning approaches for labelling features, e.g., the YouTube8M which has a vocabulary consisting of 4716 features (e.g., ‘cat’, ‘book’, ‘egg’, etc)^121^ or human action video datasets^122^. ‘Pliers’ is a tool that uses approaches like these to automatically extract features (https://github.com/tyarkoni/pliers)^123^. It is implemented in ‘NeuroScout’ (https://neuroscout.org/), a framework for providing and using these features to advance analysis of publicly available naturalistic datasets, with plans to host the NNDb.

Brain-driven annotations potentially decrease the need to annotate everything in movies. That is, the brain data itself can be used to identify movie timecodes for acquiring more detailed annotations. This allows users of the NNDb to focus on times when networks of interest are processing information, reducing the amount of movie that needs to be annotated. For example, ICA can be used to derive networks and associated independent component timecourses (as shown in Fig. 4, top). Users of the NNDb can annotate only what happens when the response is rising (or at peaks) in these timecourses in components that represent networks of interest (thus, being able to determine what the 11 individual participant networks grossly labelled as ‘Language’ are doing in Fig. 4). This can be done manually, with the aforementioned automated approaches or in a crowdsourced manner. For example, one could submit the videos from the at rise times in IC timecourses associated with Fig. 4 (top) containing the amygdala and have thousands of people quickly label observed emotional characteristics. Prior neuroscience research has successfully made use of crowdsourcing, e.g., using Zooniverse for things like quality control of image registration (https://www.zooniverse.org/projects/simexp/brain-match)^124^.

### Analysis bottleneck

Another potential bottleneck is analysis. There are arguably no standardised approaches for analysing complex and high dimensional fMRI data from long naturalistic stimuli like movies (though a few approaches are becoming increasingly common^64,125,126^). The computer science community has learned that an effective way to foster research on a topic is by running machine learning competitions on fixed datasets. These competitions allow unambiguous comparison of solutions to a problem and allow small improvements to be clearly noted and published. For example, the annual ‘ImageNet Large Scale Visual Recognition Challenge’ (ILSVRC) resulted in algorithms that outperform humans far more quickly than expected^127^. Machine learning approaches are becoming an increasingly common way to analyze fMRI data, with a growing number of examples applied to naturalistic movie stimuli^126,128^. We suggest that, to generate innovation in analysis, that competitions similar to the ILSVRC could be run using the NNDb to crowdsource the development of new machine learning (and other) approaches for fMRI data from movies. Indeed, such approaches are already being used to understand, e.g., how the visual system processes everyday objects (http://algonauts.csail.mit.edu/challenge.html).

### Future of the NNDb

We plan to make more fMRI data from the current and additional movies available as it is acquired, at the same (1.5 T) and higher field strengths (3 T), in typically developing and clinical groups. We hope that we will be able to amass the collective effort of our lab and other groups and platforms (like NeuroScout) to collate annotated stimulus features to be able to ask more and more specific questions of the data. Similarly, we hope a collective effort in analyses approaches applied to the NNDb (e.g., through machine learning competitions) will lead to advances in understanding how the brain works. We will make additional data, annotations and improvements to older annotations and code for analyses available in regular updates.

## Data Availability

Scripts used in this manuscript are available at https://github.com/lab-lab/nndb (Online-only Table 1). Additional information can be found at http://naturalistic-neuroimaging-database.org/.

## Online-only Table

**Online-only Table 1:** Location of the resources associated with the *naturalistic neuroimaging database* procedures, preprocessing and data validation steps.

Component	Subcomponent	Step	URL	File / Location
Procedure	Session 1	NIH Toolbox	https://openneuro.org/datasets/ds002837	nih_toolbox_demographics.csv
Procedure	Session 1	NIH Toolbox	https://openneuro.org/datasets/ds002837	nih_toolbox_data.csv
Procedure	Session 1	NIH Toolbox	https://openneuro.org/datasets/ds002837	nih_toolbox_scores.csv
Procedure	Session 2	Functional MRI	https://openneuro.org/datasets/ds002837	sub-<ID>/func/
Procedure	Session 2	Functional MRI	https://openneuro.org/datasets/ds002837	derivatives/sub-<ID>/func/
Procedure	Session 2	Anatomical MRI	https://openneuro.org/datasets/ds002837	sub-<ID>/anat/
Procedure	Session 2	Anatomical MRI	https://openneuro.org/datasets/ds002837	derivatives/sub-<ID>/anat/
Preprocessing	Anatomical	Skull stripping	https://github.com/lab-lab/nndb/blob/master/fMRI_preprocessing/	preprocessing_anatomical_freesurfer.sh
Preprocessing	Anatomical	MNI align	https://github.com/lab-lab/nndb/blob/master/fMRI_preprocessing/	preprocessing_anatomical_nonlinearly_align_to_mni.sh
Preprocessing	Anatomical	Inflation & segmentation	https://github.com/lab-lab/nndb/blob/master/fMRI_preprocessing/	preprocessing_anatomical_freesurfer.sh
Preprocessing	Anatomical	WM/CSF mask	https://github.com/lab-lab/nndb/blob/master/fMRI_preprocessing/	preprocessing_functional_detrend_normalize.sh
Preprocessing	Functional	Time shifting	https://github.com/lab-lab/nndb/blob/master/fMRI_preprocessing/	preprocessing_functional_slice_timing.sh
Preprocessing	Functional	Despiking	https://github.com/lab-lab/nndb/blob/master/fMRI_preprocessing/	preprocessing_functional_despike.sh
Preprocessing	Functional	Volume registration	https://github.com/lab-lab/nndb/blob/master/fMRI_preprocessing/	preprocessing_functional_volume_registration.sh
Preprocessing	Functional	MNI align	https://github.com/lab-lab/nndb/blob/master/fMRI_preprocessing/	preprocessing_functional_align_to_mni_anatomical.sh
Preprocessing	Functional	Mask timeseries	https://github.com/lab-lab/nndb/blob/master/fMRI_preprocessing/	preprocessing_anatomical_functional_masks.sh
Preprocessing	Functional	Smooth 6 mm FWHM	https://github.com/lab-lab/nndb/blob/master/fMRI_preprocessing/	preprocessing_functional_spatially_smooth.sh
Preprocessing	Functional	Detrending w/ motion, etc.	https://github.com/lab-lab/nndb/blob/master/fMRI_preprocessing/	preprocessing_functional_detrend_normalize.sh
Preprocessing	Functional	Timing correction	https://github.com/lab-lab/nndb/blob/master/fMRI_preprocessing/	preprocessing_functional_timing.sh
Preprocessing	Functional	Concatenation	https://github.com/lab-lab/nndb/blob/master/fMRI_preprocessing/	preprocessing_functional_concatenate.sh
Preprocessing	Functional	ICA artifact removal	https://github.com/lab-lab/nndb/blob/master/fMRI_preprocessing/	preprocessing_functional_ica_based_artifact_cleansing.sh
Validation	Overall Quality	Timepoint outliers	https://openneuro.org/datasets/ds002837	derivatives/sub-<ID>/motion/
Validation	Overall Quality	Maximum Movement	https://openneuro.org/datasets/ds002837	derivatives/sub-<ID>/motion/
Validation	Overall Quality	ICA artifacts	https://openneuro.org/datasets/ds002837	derivatives/sub-<ID>/func/
Validation	Overall Quality	MRIQC	https://openneuro.org/datasets/ds002837	derivatives/mriqc/
Validation	Intersubject Correlation	Correaltions - LME-CRE	https://github.com/lab-lab/nndb/blob/master/fMRI_functional/	nndb_isc.sh
Validation	Network Labelling	Annotations	https://openneuro.org/datasets/ds002837	stimuli/
Validation	Network Labelling	GLM - Group t-tests	https://github.com/lab-lab/nndb/blob/master/fMRI_functional/	nndb_network_labelling_main.sh

URLs link to publically available data and scripts/code associated with this manuscript. Text corresponds to Figure 1.
